# The Effect of Organic Matter from Sewage Sludge as an Interfacial Layer on the Surface of Nano-Al and Fluoride

**DOI:** 10.3390/molecules28186494

**Published:** 2023-09-07

**Authors:** Fan Gao, Xueqin Ma, Yi Tan, Bo Zhang, Yixing Yang, Hongqi Nie, Zhixiang Xu

**Affiliations:** 1 School of Energy and Power Engineering, Jiangsu University, Zhenjiang 212013, China; 2222206051@stmail.ujs.edu.cn (F.G.);; 2 School of Energy and Environment, Southeast University, Nanjing 210096, China; bozhang@seu.edu.cn; 3 Oil &Gas Technology Research Institute, PetroChina Changqing Oilfield Company, Xi’an 710018, China; yyx4_cq@petrochina.com.cn; 4 Science and Technology on Combustion, Internal Flow and Thermostructure Laboratory, Northwestern Polytechnical University, Xi’an 710072, China

**Keywords:** sewage sludge, energetic materials, nano aluminum powder, ball milling, proteins

## Abstract

Due to its high reactivity, the nano aluminum particle (n-Al) has attracted more attention in energetic materials but is easily oxidized during processing. In order to realize sewage sludge (SS) resource and n-Al coating, the organic matter was extracted from SS, using the deep eutectic solvent method due to its strong dissolving capacity, and then the organic matter was pretreated by ball milling, which was used as an interfacial layer between n-Al and fluoride. It was found that organic matter was successfully extracted from SS. The main organic matter is proteins. The ball milling method can effectively destroy the secondary structure of proteins to release more active functional groups. During the pretreatment, the Maillard reaction broke the proteins structure to form more active low molecular weight compounds. It was confirmed that n-Al can be coated by PBSP under mild conditions to form a uniform core-shell structure. PFOA can effectively coat the n-Al@PBSP to form n-Al@PBSP/PFOA, which can enhance the combustion of n-Al. The gas phase flame temperature can notably improve to 2892 K. The reaction mechanism between n-Al and coating was analyzed. The results could help SS treatment and provide new insights for n-Al coating and SS-based organic matter recovery and utilization.

## 1. Introduction

Modern wastewater treatment plants (WWTP) can easily remove organic pollution and nutrients from wastewater but inevitably generate a large amount of sewage sludge (SS) due to biological process wide application. SS is characterized as having about 80~85% water content and is rich in organic components, like extracellular polymeric substances (EPS), which consist of proteins, polysaccharides, DNA, lipids, glycoprotein, and humic-like substances, etc. [1,2]. Due to the presence of organic matter, it has attracted much attention in generating energy [3,4,5,6] and carbon materials [7,8]. Many researchers also focused on EPS recovery from SS with different methods [9], although the efficiency of recovery is still very low [10]. In short, the application of organic matter from SS is still of low value, resulting in no motivating force to drive SS resources for poor economies.

Recently, due to high densities, combustion enthalpy, and catalytic properties, metal particles are attractive in solid propellants, especially aluminum [11]. Aluminum powder utilization in solid propellant can notably improve the burning rate [12,13,14] and catalyze the decomposition of oxidants [15,16]. Due to its larger specific surface area and lower ignition temperature, the nano aluminum particle (n-Al) has attracted more attention in energetic materials [17,18]. However, n-Al, possessing a high specific surface area and high surface energy, can react with water and oxygen molecules in an ambient environment to form Al_2_O_3_, which will reduce the active aluminum content, and deteriorate the combustion performance of the propellant. On the other hand, the high specific surface area makes n-Al more easily agglomerated, resulting in serious negative effects for the rocket [19,20].

In order to address the issue, coating is an important method, including physical and chemical methods, which can form stable coatings on n-Al via strong chemical bonding and interfacial interactions between n-Al and coating [21,22]. Various organic matter and inorganic matter coating materials have been used to coat n-Al, especially fluorine polymer [23,24]. It was found that, in addition to preventing the oxidation of n-Al powders, the fluorine polymer shell also significantly reduced ignition delay and improved aluminum powders’ combustion efficiency [25,26]. Fluorine polymer can be used as an oxidant to enhance the combustion of n-Al. However, the interfacial interaction between n-Al and fluorine polymer is still weak due to the weak interfacial interactions [27]. It has been found that the oxidative polymerization of dopamine can form polydopamine (PDA) coatings on n-Al under mild conditions [28]. Dopamine possessing catechol and amine groups can form strong bonds with n-Al, including covalent and hydrogen bonds. Hence, enhancing interfacial interactions via covalent and hydrogen bonds becomes an important issue for improving coating stability, especially via simple approaches to realize fluorine coating.

Although many methods have been offered to recover EPS from SS, to date, no more promising method has been proposed. How to utilize organic matter in SS has become a hot topic for its huge volume and potential damage to the environment and humans. As is well known, proteins and polysaccharides are the dominate components in EPS [29], which has more reaction activity at low temperatures for its rich –NH_2_ and –OH. Due to their unique structure, parts of protein in EPS show dissolution in water, especially loosely bound EPS, while others are indissoluble in water [30,31]. Therefore, developing a relatively simple approach to recover EPS and enhancing the EPS dissolution in water is very important to realize SS resources.

Deep eutectic solvents (DES) consist of hydrogen bond acceptors (HBAs) and hydrogen bond donors (HBDs) [32], and have advantages such as renewability, biodegradability, non-toxicity, low volatility, recyclability, and large-scale use [33]. DES has been widely used in material synthesis, biomass processing, and many other fields based on its excellent properties [34,35]. Previous studies have shown that DES can significantly disrupt the EPS of SS via promoting protein hydrolysis [36,37]. Therefore, this provides a possibility for separating the organic matter from SS with DES.

Here, a promising method is described to recover organic matter from SS. This study utilizes alkaline DES to promote protein hydrolysis to disrupt the EPS of SS and release organic matter for achieving the goal of the efficient separation of organic matter from SS. The organic matter is used to coat nano-Al as an interfacial layer. It can further be coated by fluoride. This strategy leads to the enhancing reaction between n-Al and organic matter and further improves intermolecular interactions, especially the Mailland reaction between the protein and polysaccharide. It can provide more anchor points to graft fluoride. This strategy offers new insights for n-Al coating and SS-based organic matter recovery and utilization.

## 2. Results

### 2.1. Characteristics of PBSP

In order to analyze the chemical components of PBSP, PBSP-3000 and PBSP-10000 were analyzed firstly by elemental analysis, as shown in Table 1. It has been found that nitrogen content in PBSP exceeded 8%, and sulfur was found (>0.5%), indicating PBSP containing proteins. The difference between PBSP-3000 and PBSP-10000 in elemental content was small. Furthermore, the oxygen content in PBSP was very high, indicating that PBSP also contained polysaccharides. It was confirmed that there is high protein content in PBSP. In order to demonstrate the content of proteins in PBSP, the content of polysaccharides and proteins were analyzed. It was found that the polysaccharide was 22.92% and protein was 75.25%. The ash in PBSP was 0.8%.

**Figure 1:**
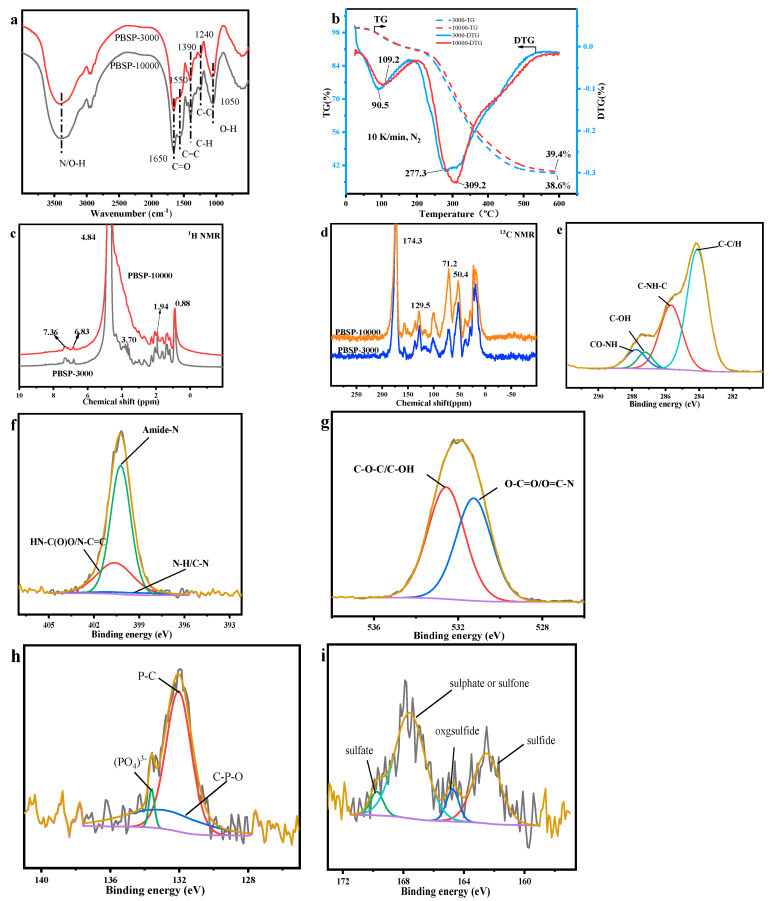
The properties of PBSP. (**a**) FTIR results; (**b**) TG-DTG results; (**c**,**d**) NMR results; (**e**–**i**) XPS results.

FTIR spectroscopy was also used to reveal another clue to the functional groups of the PBSP (Figure 1a). The strong peak located at 3400 cm^−1^ was ascribed to the stretching vibration of N–H and O–H in proteins and polysaccharides. Another band at 1650 cm^−1^ (amide I stretching vibration of C=O in proteins) and 1050 cm^−1^ (stretching vibrations of C–O in polysaccharides) were also strong, indicating that proteins and polysaccharides are the main components in PBSP. Another strong peak located at 1390 cm^−1^ was related to the symmetric stretching vibration of COO^−^ [38]. Another possibility was C–H vibration. The weak band at 1550 cm^−1^ was related to C=C vibration in aromatic compounds. The weak peak located at 1240 cm^−1^ probable was stretching vibrations of C–N of proteins in PBSP.

TG was used to analyze the thermal decomposition properties of different molecular PBSP weights (Figure 1b). The first sharp peak was found at about 100 °C, indicating that PBSP has strong hygroscopicity. About 10% of water was removed from TG curve. Another sharp peak was near 300 °C. The peak temperature of PBSP-3000 was about 280 °C, while PBSP-10000 was about 310 °C. It indicated that high molecular weight PBSP has a high thermal decomposition temperature. The difference in the TG curves indicated that low molecular weight compounds existed in the PBSP-3000. The residue of PBSP-3000 was 38.6%, while PBSP-10000 was 39.4%. From TG and DTG curves, it was confirmed that high molecular weight PBSP has better thermal stability.

In order to further investigate the properties of PBSP, NMR was used to analyze the PBSP structure (Figure 1c,d). In ^1^H NMR of PBSP, a very sharp peak located at 4.8 ppm was probably ascribed to polysaccharide in PBSP. A very broad peak in the range of 3~4.5 ppm was related to amide in proteins. Before 2 ppm, it was a proton of aliphatic groups. Weak peaks can also be found in the range of 2~3 ppm, which was ascribed to the proton of unsaturated hydrocarbons. Weak peaks can also be found near 7 ppm, which belonged to the aromatic ring. The ^13^C NMR results found a very sharp peak at about 174 ppm ascribed to carboxylic acid [39], indicating that PBSP contains proteins. In the range of 0~70 ppm, the peaks were corresponded to the aliphatic region. Sharp peaks located at 100 and 129.5 ppm belonged to sp^2^ carbon atoms. The relatively weak peak located at 157 ppm was ascribed to an amide. In the range of 100~160 ppm, the peaks belonged to unsaturated carbon atoms, including aromatic and alkenes carbon.

XPS analysis shows the heteroatom’s functional groups properties (Figure 1e–i). After fitting, four main C1s peaks reveal chemical components contributing to the spectra, namely C–C/C–H (283.6 eV), C–N (285.6 eV), C–O–H (287.2 eV), and CO–NH (287.7 eV). The main peak was C–C/C–H (283.6 eV) and C–N (285.6 eV) [40]. And CO-NH was also found, indicating that PBSP contains protein. Three N1s peaks were observed in the spectra of PBSP and assigned as N–H/C–N bond (398.7 eV), amide N (400.5 eV), and HN–C(O)O/N–C=C (401.2 eV) [41]. The main peak was amide N, indicating that the main component in PBSP was protein. Two peaks can be found in the O1s peak. The first one was attributed mainly to oxygen forming a double bond with carbon (O–C=O and O=C–N) at 531.1 eV, and the second one to single bond oxygen with hydrogen or carbon (C–OH and C–O–C) at 532.4 eV [42]. The content of the two peaks has no notable difference, indicating the high content of the C–O bond in PBSP for the presence of polysaccharide. Three peaks can be found in the P1s peak, the sharp peak was P–C (132.1 eV), the second peak was PO_4_^3−^ (133.5 eV), and P–O (133.2 eV) [43]. This indicated that, after DES treatment, organic phosphorus was released from SS. Two main peaks can be found in the S1s peak, one was sulfate (167.6 eV), and another was sulfide (162.5 eV) [44]. This means that organic sulfur can be found in PBSP. This was in line with the elemental analysis results. The XPS results also confirmed that PBSP contains proteins. It has some different XPS results [45], indicating that polysaccharides in PBSP influence the structure of the protein. The contents of difficult functional groups are listed in Table S1.

### 2.2. After Ball Milling for PBSP

**Figure 2:**
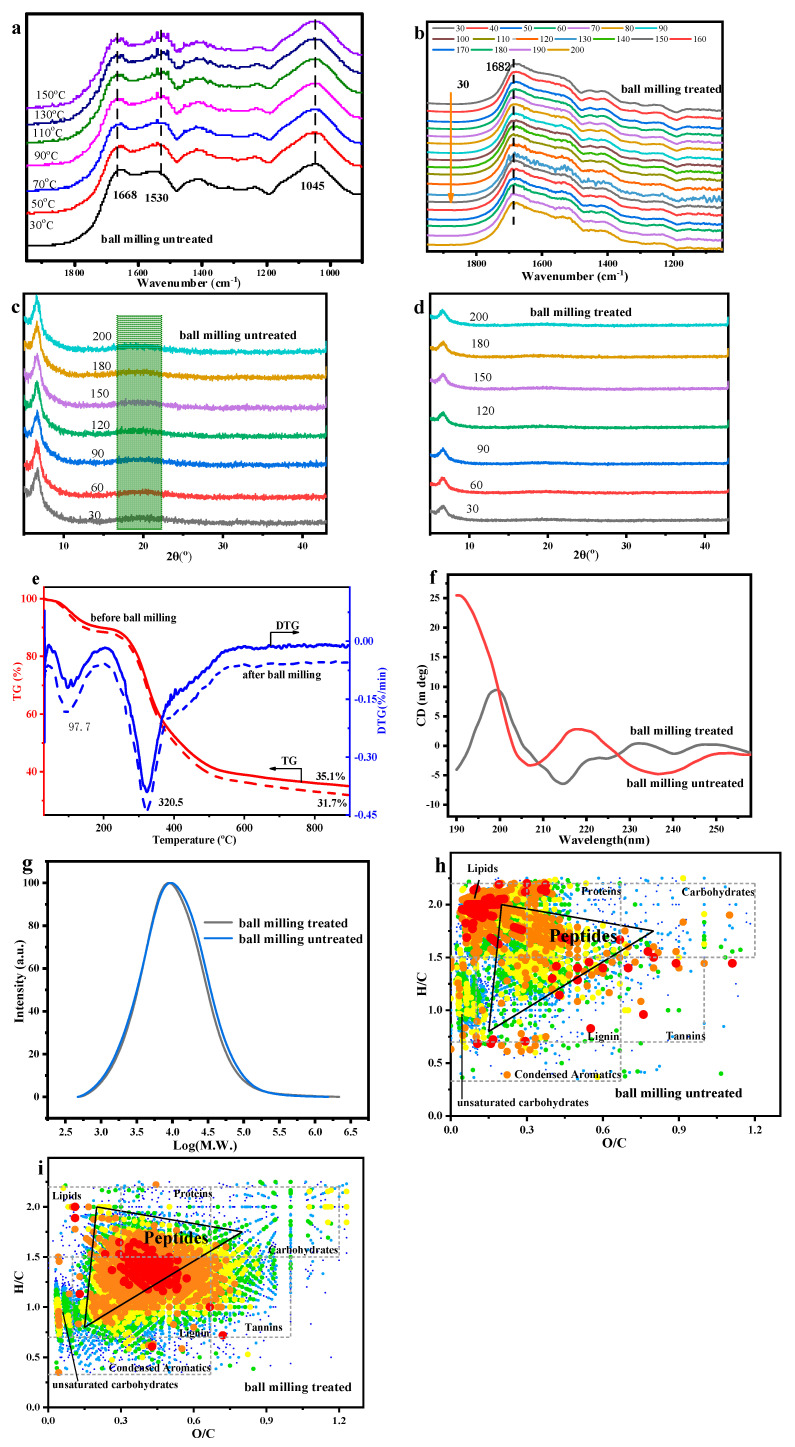
The influence of ball milling treatment on PBSP. (**a**) In situ FTIR results of untreated PBSP; (**b**) In situ FTIR results of treated PBSP; (**c**) XRD results of untreated PBSP; (**d**) XRD results of treated PBSP; (**e**) TG-DTG results of PBSP with and without ball milling treatment; (**f**) CD spectra; (**g**) Molecular distribution with and without ball milling treatment; (**h**) ESI FT-ICR MS results of ball milling untreated; (**i**) ESI FT-ICR MS results of ball milling treated.

In order to further explore the probe PBSP dissolution possibility, ball milling was carried out as a pretreatment to enhance the dissolution of PBSP in water first. Here, PBSP-10000 was selected. The FTIR with and without ball milling with different temperatures was first analyzed (Figure 2a,b). In the Amide I band of the protein (1700–1600 cm^−1^), a sharp peak can be found, which arises from the vibration of the C=O stretching of the protein backbone. This was strictly related to the protein secondary. The detailed information was that the region of 1668–1680 cm^−1^ can be assigned to β-turn conformations, while the peak located at 1659 m^−1^ and 1646 cm^−1^ can be attributed to α-helix and random structures, respectively [46]. The peaks located at 1691 cm^−1^, 1638 cm^−1^, and 1628 cm^−1^ were assigned to β-sheet structures, whereas that of low intensity at 1620 cm^−1^ can be assigned to intermolecular β-sheet aggregates [47,48]. Compared to with and without ball milling, it was found that, after ball milling, in the second derivative spectrum of PBSP, there was a decrease in the intensity of β-sheet structures and α-helix, while β-turn conformations had a notable increase. The information on secondary structure content is listed in Table S2. However, compared to FTIR results with and without ball milling, there was a great difference with the peak located at 1050 cm^−1^, which was ascribed to the glucosidic bond of polysaccharide in PBSP. This indicated that polysaccharide, during ball milling, underwent a chemical reaction, resulting in glucosidic bond disappearance. From the PBSP components, the possibility reaction was the Maillard reaction, which was ascribed to the reaction between proteins and polysaccharides.

In the XRD pattern of the PBSP with and without ball milling, the notable peak was 6.65° and 20.20° (Figure 2c,d). In other words, the polysaccharide in PBSP, and without the purification of PBSP, influenced the protein structure. After ball milling, the *β-*sheet structure almost disappeared, indicating that the secondary structure of proteins was destroyed after ball milling. It was confirmed by the FTIR results that a reaction occurs during ball milling. Increasing temperature was not a key factor.

In order to further analyze the influence of ball milling on PBSP, a thermal analysis of PBSP was carried out (Figure 2e). The first sharp peak was found at about 100 °C, indicating that PBSP has strong hygroscopicity. In other words, it was confirmed that PBSP contained polysaccharides. About 10% of water was removed according to TG curves. It was also found that, after ball milling treatment, PBSP has a stronger hygroscopicity ability than untreated PBSP for exceeding 2% water removal in TG. Another sharp peak was near 320 °C, indicating that the thermal decomposition of proteins and polysaccharides occurred [49]. The residue of PBSP was 35.1% and 31.7% of untreated PBSP and ball-milling-treated PBSP, respectively. From the TG and DTG curves, no notable difference can be found.

To better understand the impacts of ball milling, the secondary structure of proteins with CD spectra was investigated (Figure 2f), which is a sensitive and well-established technique for characterizing the secondary structure of proteins in a solution [50]. It was found that, without a ball milling sample, the negative peak at 206 nm and negative shoulder in the range of 220–250 nm were indicative of the β-sheet structure [51]. After ball milling, a sharp positive peak at 200 nm (random coil) and a weak negative peak at 214 nm (indicating β-sheet character) were found [52]. The negative peaks at 206 and 230 nm were reported to be caused by a negative Cotton effect [53]. According to the CD spectrum, it was found that ball milling has a significant influence on the PBSP component.

The average molecular weight of PBSP was also measured (Figure 2g). After ball milling, the average molecular weight of PBSP decreases to 85,843 from 154,999, indicating that ball milling destroyed the molecular structure to form low molecular weight compounds. This confirmed that ball milling has a significant influence on PBSP.

In order to further analyze the influence of ball milling, ESI FTICR MS was used to analyze the components change after ball milling (Figure 2h,i). It was found that the content of peptides after ball milling has a notable increase, indicating that more proteins were broken into relatively low molecular weight peptides. In other words, PBSP has carried out a reaction during ball milling, especially the Maillard reaction.

**Figure 3:**
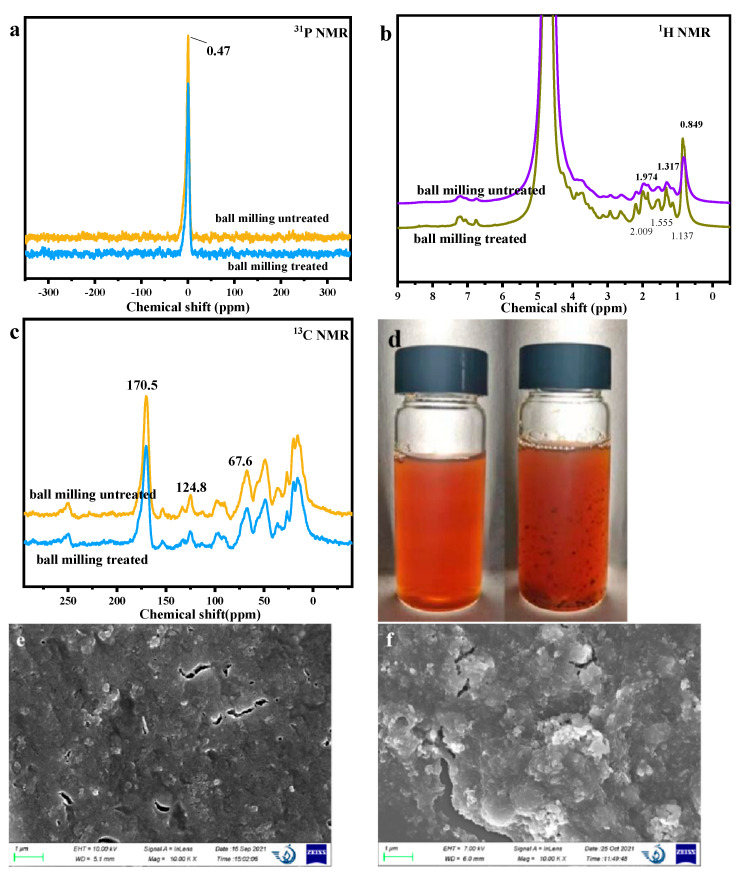
The influence of ball milling treatment on NMR and surface properties for PBSP. (**a**) ^31^P NMR results; (**b**) ^1^H NMR results; (**c**) ^13^C NMR results; (**d**) The solution of PBSP with and without ball milling treatment; (**e**) SEM of untreated PBSP; (**f**) SEM of treated PBSP.

To explore the change in the ball milling, NMR was also used to analyze the chemical structure change (Figure 3a–c). No change can be found in the ^31^P NMR. The peak at 0.47 ppm was ascribed to phosphate, indicating the cell wall destruction during DES treatment to release organic phosphorus. In the ^13^NMR and ^1^H NMR results, it was found that almost no notable difference could be found, indicating that the main chemical structure has no change.

In order to further analyze the influence of ball milling on PBSP, the dissolution of PBSP with and without ball milling was used for analysis (Figure 3d). It was found that, after ball milling treatment, PBSP can be uniformly dissolved to form a transparent solution, while it was a muddy solution without ball milling treatment of PBSP and particles that can be found in the solution. The morphology of PBSP with and without ball milling was investigated (Figure 3e,f). It was observed that the surface structure of the untreated PBSP was compact. Some pores and cracks can be found on the surface of PBSP. The treated PBSP was broken, rough, and looked like unconsolidated and flaky fragments. This indicated that ball milling could damage the surface structure.

### 2.3. The Properties of n-Al@PBSP and n-Al@PBSP/PFOA

**Figure 4:**
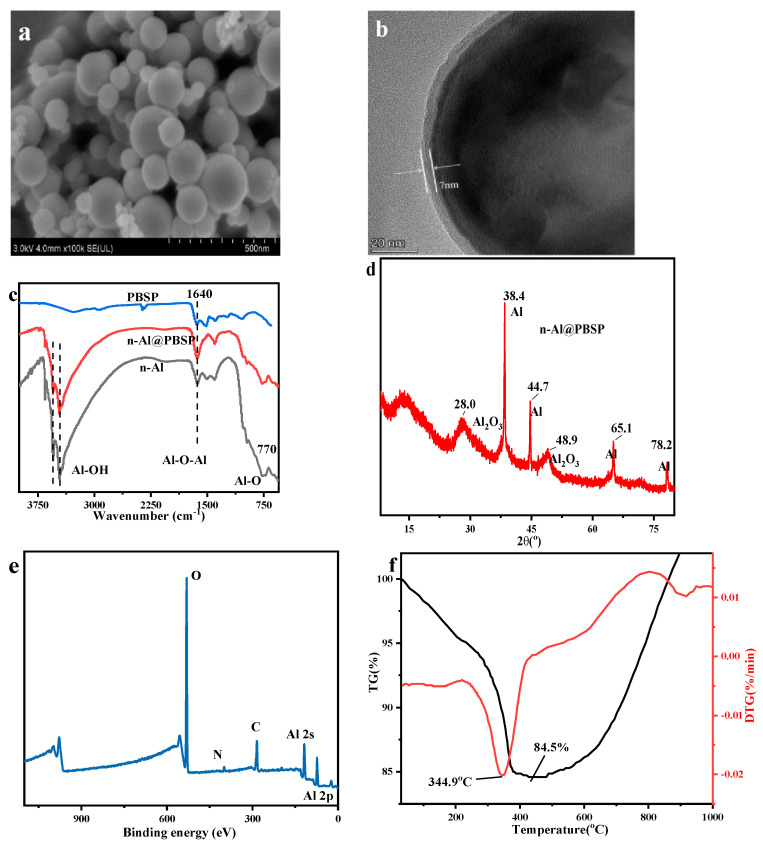
The properties of n-Al@PBSP. (**a**) SEM of n-Al@PBSP; (**b**) TEM of n-Al@PBSP; (**c**) FTIR results of samples; (**d**) XRD results of n-Al@PBSP; (**e**) XPS of n-Al@PBSP; (**f**) TG-DTG results of n-Al@PBSP.

The n-Al@PBSP particles were obtained simply by the one-step graft copolymerization of PBSP on the surface of n-Al in water at 60 °C for 1 h. The –NH_2_ and –OH of PBSP can remarkably promote the cross-linking reaction between PBSP and n-Al, resulting in the acceleration of the coating formation process. Figure 4a,b showed SEM and TEM images of the Al@PBSP. The interaction between the PBSP and n-Al mainly depended on their functional groups, resulting in n-Al/PBSP being quite dense and uniform with a thickness of ~7 nm due to the high activation of PBSP. The uniform core-shell structure can be found via SEM. Hence, n-Al can be coated by PBSP under mild conditions.

The surface chemical functional groups of the n-Al@PBSP were analyzed by FTIR (Figure 4c) and XPS (Figure 4e). The broad band near at 3500 cm^−1^ was ascribed to the –OH groups. The absorption band appeared at 1640 cm^−1^ and belonged to Al–O–Al vibration. The broad peak appearing at 770 cm^−1^ was due to Al–O stretching vibrations. On the other hand, the typical characteristic peak located at 1030 cm^−1^ belonging to polysaccharide C–O vibration disappeared in n-Al@PBSP, indicating that the reaction between n-Al and PBSP occurred. After coating, the typical peak of n-Al still can be found in FTIR, indicating that PBSP was coated in the surface of n-Al, not mixed of solid samples. Meanwhile, after coating, XRD results (Figure 4d) showed that the characteristic peaks at 2θ = 38.4°, 44.7°, 65.1°, and 78.2°, were clearly observed on the n-Al@PBSP, assigned to the (111), (200), (220), and (311) crystal planes of Al crystals, respectively. The 18.0° and 48.9° were ascribed to Al_2_O_3_ on the surface of n-Al. This result revealed that, during coating, PBSP would not destroy the crystalline structure of nano-Al particles. The XPS results (Figure 4e) in n-Al@PBSP mainly displayed O 1s (532.7 eV), C 1s (284.9 eV), N 1s (400.0 eV), Al 2s (117.9 eV), and Al 2p (72.9 eV) peaks. The C 1s, N 1s, and O 1s peaks that appeared were mainly attributed to PBSP. The sharp Al 2s and Al 2p peaks that appeared indicated that the n-Al@PBSP still has a high Al content. The Al–O can be found in split peaks. The detailed information can be found in Figure S1.

TG-DTG revealed the thermal stabilities of the n-Al@PBSP under N_2_ atmosphere (Figure 4f). Before 200 °C, almost no peak can be found. Compared to the thermal decomposition of PBSP, this indicated that hydrophilicity functional groups on the PBSP reacted with –OH on the surface of n-Al. The sharp peak was located at 344.9 °C, which was ascribed to PBSP thermal decomposition. It was slightly higher than the pure PBSP peak temperature. The mass loss was about 14% at this temperature. Actually, the amount of PBSP and citric acid was 8%. This indicated that about 6% n-Al or Al_2_O_3_ on the surface of n-Al took part in the reaction. Combined with peak temperature and mass loss, this was probably related to citric acid, which can catalyze PBSP thermal decomposition and react with Al_2_O_3_. After 680 °C, mass loss decreased rapidly. This was related to the reaction between N_2_ and n-Al. According to the above, it was confirmed that PBSP can effectively coat the n-Al and enhance the thermal stability of the n-Al.

**Figure 5:**
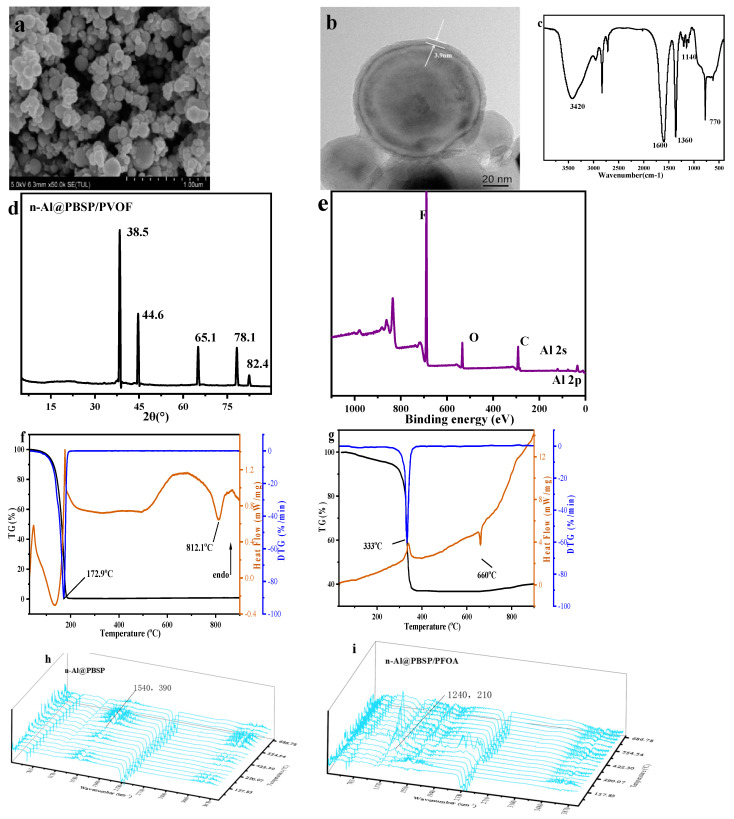
The properties of n-Al@PBSP/PFOA. (**a**) SEM of n-Al@PBSP/PFOA; (**b**) TEM of n-Al@PBSP/PFOA; (**c**) FTIR result of n-Al@PBSP/PFOA; (**d**) XRD results of n-Al@PBSP/PFOA; (**e**) XPS of n-Al@PBSP/PFOA; (**f**) TG-DSC-DTG results of PFOA; (**g**) TG-DSC-DTG results of n-Al@PBSP/PFOA; (**h**) TG-FTIR results of n-Al@PBSP; (**i**) TG-FTIR result of n-Al@PBSP/PFOA.

In order to further improve the properties, PFOA was also further coated in n-Al@PBSP. The n-Al@PBSP/PFOA particles were obtained. In fact, perfluoroalkyl acid has been used to coat n-Al [54,55,56]. Due to the low content of anchor sites on the surface of n-Al, it still needed harsh conditions in order to coat. Due to there being more anchor sites on the surface of n-Al@PBSP, it can be prepared in water. Figure 5a,b showed SEM and TEM images of the Al@PBSP/PFOA. Parts of aggregation can still be found. The strong polarity of C–F can form hydrogen bonds with H–O/N of PBSP. And –COOH in PFOA can also react with –OH in citric acid and PBSP during heating to form ester, resulting in n-Al@PBSP/PFOA being quite dense and uniform with a thickness of ~4 nm. Compared to n-Al@PBSP, due to the strong acid properties of PFOA, it would hydrolyze parts of PBSP, resulting in the thickness decreasing. The uniform core-shell structure can be found via TEM. Hence, PFOA can be coated in n-Al@PBSP under mild conditions to form n-Al@PBSP/PFOA.

The surface chemical functional groups of the n-Al@PBSP/PFOA were analyzed by FTIR (Figure 5c). The sharp peak located at 1600 cm^−1^ belonged to the Al–O–Al vibration. Another sharp peak appearing at 770 cm^−1^ was due to Al–O stretching vibrations. The sharp peak located at 1360 cm^−1^ was C–F of PFOA. The weak peaks located about 1140 cm^−1^ were CF_2_. The XRD results showed that characteristic peaks at 2θ = 38.4°, 44.7°, 65.1°, and 78.2°, were clearly observed on the n-Al@PBSP/PFOA, assigned to the (111), (200), (220), and (311) crystal planes of Al crystals, respectively (Figure 5d). F 1s (687.9 eV), O 1s (532.7 eV), C 1s (284.9 eV), Al 2s (117.9 eV), and Al 2p (72.9 eV) peaks clearly appeared (Figure 5e), while N 1s (400.0 eV) almost disappeared. The SEM-EDS results showed that the atom content of N was 1.49% (Figure S2). This indicated that PFOA has an important influence on the stability of PBSP. The peaks of Al 2s and Al 2p were also very weak. The SEM-EDS results showed that the atom content of Al was 47.30%. The FTIR, XPS, and SEM-EDS results demonstrated that PFOA has, indeed, been present at the surface of the particle and bounded to the n-Al@PBS surface.

Due to the sublimation of PFOA before 150 °C, mass loss increased stepwise after 100 °C [56,57]. The TG-DTG-DSC of PFOA under N_2_ with 20 k/min is listed in Figure 5f. It was found that, before 180 °C, the PFOA was sublimated completely, indicating that during the heating process, PFOA would first sublimate before the thermal decomposition of n-Al@PBSP/PFOA. The gas PFOA would be blown away by N_2_, which would influence the exothermic reaction of n-Al@PBSP/PFOA. According to Figure 4f, PBSP can also decompose near 280 °C. After 300 °C, the decomposition temperature of PFOA began. The disassociation of carboxyl promoted initial decomposition and the appearance of the exothermic peak. On the other hand, due to the endothermic reaction of thermal decomposition, PFOA’s boiling point and decomposition temperature are very close, leading to a weak exothermic peak appearing [54]. It is worth noting that, due to the high reactivity of fluorocarbon free radicals, it can react with n-Al easily, indicating that the main cleavage at this stage was the C–C bond, not the C–F bond. This revealed that the carboxyl significantly influenced the initial interaction. However, the very sharp peak appeared at 330 °C (Figure 5g). The mass loss can reach 63%, indicating that n-Al took part in the reaction due to the low coating of 10%. Hence, regarding the exothermic peak, it was not only the coating that carried out the reaction, but n-Al also took part in the reaction. This was considered as a Pre-Ignition Reaction (PIR), which was associated with the reaction between Al_2_O_3_ and fluorine polymer, probably generating AlF_3_ [54,58] and exiting on the surface of the Al powders [59]. Hence, the PIR process corroded the alumina shell on the surface of the aluminum powders, resulting in a more sufficient Al-fluorine reaction in the lower temperature stage. Hence, the mass loss exceeded 63%. The last endothermic peak at 660 °C resulted in the melting of n-Al due to the relatively low content of PFOA.

The TG-FTIR results of of n-Al@PBSP and n-Al@PBSP/PFOA are listed in Figure 5h,i. Compared to Figure 5h,i, it was found that weak characteristic peaks of PFOA appeared, indicating that, after 150 °C, PFOA started to sublimate. After 300 °C, the signal was very strong and a CO peak appeared, indicating that PFOA began to decompose. Active C-F free radicals can react with Al, resulting in PIR, an exothermic peak appearing, and mass loss aggravation. Hence, the TG-FTIR (Figure 5h,i) and TG-DSC (Figure 5f) results can explain why the endothermic peak of Al appears in Figure 5g.

**Figure 6:**
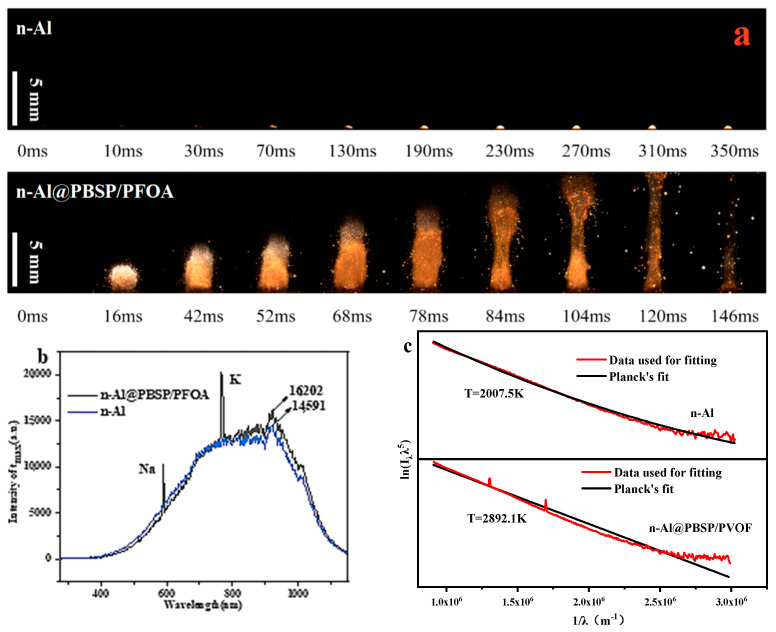
Combustion behaviors of the samples. (**a**) Combustion behaviors of the samples with and without coating; (**b**) Maximum flame intensity spectra; (**c**) Gas phase flame temperature.

To investigate the influence of coating on the combustion of n-Al powder, the combustion behaviors of samples with and without coating are shown in Figure 6a. When the laser hit the n-Al powder under N_2_, it was ignited and began to burn after 70 ms. This process showed obvious Al melting, indicating that the combustion of n-Al powder occurred after it melted. In total, the flame was very weak. When n-Al@PBSP/PFOA was ignited, at 16 ms it showed an obvious sputtering flame of n-Al particles, which was the diffusion combustion stage. In this stage, the burning particles were thought to be mainly n-Al particles because n-Al particles have high reactivity, lower ignition temperature, and a faster burning rate, which can be easily ignited. After 42 ms, the flame’s area and brightness increased rapidly, which was a typical stable combustion stage. In the stable combustion stage, the flame brightness and the area of the n-Al@PBSP/PFOA sample obviously became larger, indicating that the combustion intensity of the n-Al@PBSP/PFOA became strong. According to the results of Figure 6a, it was confirmed that a PBSP/PFOA coating has a notable influence on the n-Al combustion.

The combustion intensity of the samples was also analyzed. When the n-Al was coated by PBSP/PFOA, the combustion intensity of the n-Al samples was enhanced due to the presence of PFOA (Figure 6b). The Al oxide layer was destroyed by the PFOA at a low temperature (Figure 5g), which strengthened the reaction between the internal active Al and the external gas. It is worth noting that the fluorination combustion reaction between Al and fluoride released more heat than the oxidation combustion reaction [60]. Hence, the combustion intensity of the n-Al@PBSP/PFOA was enhanced. The gas phase flame temperature of n-Al@PBSP/PFOA was 2892 K, while n-Al was 2007 K. Hence, adding PFOA can notably enhance the n-Al combustion performance.

## 3. Discussion

For the presence of proteins in PBSP, destroying the secondary structure of proteins to release more active sites becomes very important for PBSP utilization as an interfacial layer. The components in PBSP have mainly included polysaccharides (22.92%) and proteins (75.25%), as shown in the above analysis results. The reaction between polysaccharides and proteins can occur at a relatively high temperature via the Maillard reaction. Ball milling was used as a pretreatment method to obtain a uniform PBSP solution, which can provide more –NH_2_ and –OH groups from PBSP to anchor the n-Al. Due to the presence of –OH on the surface of n-Al, –NH_2_ and –OH groups from PBSP can react to dehydration, which promoted the formation of the dense coating.

As is well known, β-sheets played a fundamental role in enhancing mechanical properties. It was demonstrated that the content of β-sheets in PBSP notably decreased after ball milling. This indicated that a partial secondary structure probably unfolded during ball milling to participate in the Maillard reaction. Then, in the citric acid–water binary mixture system, high temperatures further promoted the secondary structure of unfolded proteins and the Maillard reaction occurring between the proteins and polysaccharide. And, also, proteins can react with fatty acids with hydrogen bonds. Hence, the citric acid–PBSP system can form a dense coating during the coating process.

Due to the low content of –OH on the surface of n-Al, fluorine polymer also has low active functional groups, resulting in harsh conditions to coat n-Al with fluorine polymer. Hence, enhancing activity sites from the interfacial layer became an important step. The above analysis showed that PBSP was a good interfacial layer material due to its rich activity sites. Hence, PFOA can be effectively coated on the surface of n-Al@PBSP to form n-Al@PBSP/PFOA. Therefore, the activity site on the interfacial layer was a significant factor.

In conclusion, this paper was the first to successfully prepare n-Al@PBSP/PFOA using proteins and polysaccharides as a feedstock from SS. It offered new insights for n-Al coating and SS-based organic matter recovery and utilization.

## 4. Materials and Methods

### 4.1. Materials

SS was supplied by the Gujing group wastewater treatment plant located in Bozhou, Anhui Province, China. The moisture and ash contents of SS (dry matter) are 85.2% and 38.7%, respectively. Perfluorooctanoic acid (PFOA), citric acid, and other chemicals are analytical grade and were directly used. The nano-Al powder is 70 nm (Zhongkeyannuo New Material Technology Co., Ltd., Beijing, China).

### 4.2. Experiment Detailed Information

(a) Protein-based mixture extracting and separation

Typically, KOH and urea were dispersed in H_2_O. The amount of KOH and urea was 3.5 mol/L and 0.6 mol/L, respectively. The KOH and urea were directly poured into water to obtain a deep eutectic (DES) solution after mixing well until transparent. Then, 5 L of DES and 5 kg of SS were mixed and soaked for 24 h, in which DES was used as a solvent to extract organic matter in SS. After 24 h, the liquid component was separated using a nylon filter cloth (1000 mesh) to obtain a low solid phase content solution. Then, the solution was repeated two times to further decrease the solid phase content. At last, the peristaltic pump (Huiyu-WT’600J-1A, HuiyuWeiye (Beijing, China) Fluid Equipment Co., Ltd.) was used to further separate the solid phase, KOH, urea, and other low molecular compounds in the solution. The different kinds of hollow cellulose nano filtration membranes (003w, Beijing Xubang Membrane Equipment Co., Ltd., Beijing, China) in the peristaltic pumps were used to trap organic matter. The hollow cellulose nano filtration membranes-10,000 was used to trap the exceeding 10,000 Da organic matter in solution. When the pH value of the solution reached 7, the solution stopped the separation. The obtained protein-based solid product was named “PBSP-10000”. Another was PBSP-3000. And the separation solution was then heated to obtain PBSP solid at 100 °C.

(b) Ball milling process

The drying PBSP was performed using planetary ball milling equipment (Nanjing Chi Shun Technology Development Co., Ltd. PMQW2, Nanjing, China). Each experiment agate jar (100 mL) not only contained 10 g PBSP but also contained agate balls (2 big balls (ø20 mm), 10 mid-balls (ø10 mm), and 60 small balls (ø6 mm)). The ball milling condition was 500 rpm and 1 h at room temperature.

(c) n-Al coating with PBSP

After ultrasonic treatment, 0.1 g of ball milling PBSP was added to 9.9 g of deionized water to obtain the PBSP solution (1%) (Bandelin, HD2070, 70 W). An amount of 0.1 g of citric acid was added to 9.9 g of deionized water to obtain a citric acid solution (1%) after ultrasonic treatment. An amount of 4 g of PBSP solution was added to 16 g of deionized water and was kept at 90 °C for 60 min with ultrasonic treatment. Then, n-Al was added to this solution to coat, at 40 °C for 60 min, by mechanical agitation with 500 r/min. After coating, the solid was rinsed with deionized water 5 times. The solid was heated at 40 °C to obtain n-Al@PBSP.

(d) n-Al@PBSB/PFOA

Amounts of 1 g of n-Al@PBSP and 0.1 g of PFOA were added to 20 mL of deionized water in a stainless steel reactor. The mixture was mixed uniformly with mechanical agitation with 500 r/min at 60 °C for 60 min. The mixture was centrifugally separated at 8000 r/min. Then, it was rinsed with deionized water to neutralize, then solid was heated at 40 °C to obtain n-Al@PBSB/PFOA.

### 4.3. Characterization

The elemental components were analyzed using an elemental analyzer (Flash2000 CHNS). The functional groups were analyzed using Fourier transform infrared spectroscopy (FTIR, VERTEX 70 FT-IR spectrometer Bruker, Mannheim, Germany). The thermal decomposition characteristic was analyzed via Thermogravimetric analysis (TG) (TGA/DSC 3+, Mettler Toledo, Greifensee, Switzerland). N_2_ was used as a carrier gas at 20 mL/min. The crystallinity properties of the samples were analyzed using an X-ray diffraction spectrometer (XRD, D8 Advance, Bruker). The XRD with different temperatures also used the same system. The morphology of the sample was probed to ascertain the surface characteristics using a scanning electron microscope (SEM, Merlin, Zeiss, Oberkochen, Germany) and transmission electron microscope (TEM, JEM 2100F, JEOL).The carbon-containing functional groups, hydrogen-containing functional groups, and phosphorus-containing functional groups were analyzed via Nuclear magnetic resonance (NMR) analysis, including ^13^C NMR, ^1^H NMR, and ^31^P NMR spectra (400 MHz, Bruker AVANCE III 600 M). X-ray photoelectron spectroscopy (XPS, Thermo ESCALAB 250 instrument) was used to probe the surface functional groups. The Circular Dichroism spectra of the sample were measured at 190–250 nm and 25 °C using a CD spectropolarimeter (JASCO J-815, Tokyo, Japan). The molecular weight distribution of the sample was analyzed by the Shimadzu Gel Permeation Chromatography system. The ignition test of the samples was conducted according to reference [61]. The ignition system included a laser controller, a combustion chamber with transparent window, a spectral acquisition device, and a portable data collector. The components change due to ball milling was analyzed by Fourier transform ion cyclotron resonance mass spectrometry coupled with electrospray ionization (ESI FT-ICR MS), for which detailed information was provided in our previous work [62]. The operating conditions for negative-ion formation were 4.0 kV of emitter voltage, 4.5 kV of capillary column introduce voltage, and 320 V of capillary column end voltage.

## 5. Conclusions

This paper reported on organic matter extracted from SS. Then, the organic matter was pretreated via ball milling to obtain PBSP, which was used as an interfacial layer between n-Al and fluoride. The following conclusions can be drawn:

(1) The organic matter was successfully extracted from SS using the DES (KOH and urea) method.
(2) The ball milling method can effectively destroy the secondary structure of proteins to release more active functional groups. During the pretreatment, the Maillard reaction broke the proteins’ structure to form more active low molecular weight compounds.
(3) It was confirmed that n-Al can be coated by PBSP under mild conditions to form a uniform core-shell structure. PFOA can effectively be coated on the surface of the n-Al@PBSP to form n-Al@PBSP/PFOA, which can improve the gas phase flame temperature.
(4) The reaction mechanism between n-Al and the coating was analyzed.

## Figures and Tables

**Table 1:** Elemental analysis results of PBSP.

Samples	N	C	H	S	O *
PBSP-3000	8.97	38.8	5.427	0.717	46.086
PBSP-10000	8.06	38.96	5.469	0.883	46.628

* O=100-N-C-H-S5.

## Data Availability

Not applicable.

## Supplementary Materials

The following supporting information can be downloaded at: https://www.mdpi.com/article/10.3390/molecules28186494/s1, Figure S1: The results of XPS Al@PBSP. Figure S2: n-Al@ABSE@PVOF SEM-EDS. Figure S3: The results of XPS Al@PBSP/PFOA. Table S1: The content of difficult functional groups. Table S2: The content of secondary structure.

## Sample Availability

Not available.
