# Supplementary material for: The Effect of Organic Matter from Sewage Sludge as an Interfacial Layer on the Surface of Nano-Al and Fluoride
Source: Molecules. 2023 Sep 7;28(18):6494. doi: 10.3390/molecules28186494 (PMC10536677; doi:10.3390/molecules28186494)
Supplement: Supplementary file 1 [file molecules-28-06494-s001.zip › molecules-2589345-supplementary.pdf]

Supplementary Materials

The Effect of Organic Matter from Sewage Sludge as an Interfacial Layer on the Surface of Nano-Al and Fluoride

Fan Gao [1], Xueqin Ma [1], Yi Tan [1], Bo Zhang [2], Yixing Yang [3], Hongqi Nie [4] and Zhixiang Xu [1,*]

[1] School of Energy and Power Engineering, Jiangsu University, *Zhenjiang* 212013, China; 2222206051@stmail.ujs.edu.cn (F.G.)
[2] School of Energy and Environment, Southeast University, Nanjing 210096, China; bozhang@seu.edu.cn
[3] Oil &Gas Technology Research Institute, PetroChina Changqing Oilfield Company, Xi'an 710018, China; yyx4_cq@petrochina.com.cn
[4] Science and Technology on Combustion, Internal Flow and Thermostructure Laboratory, Northwestern Polytechnical University, Xi'an 710072, China
* Correspondence: xuzx@ujs.edu.cn

Table S1. The content of difficult functional groups.

C(%)	C-C/H	C-NH-C	C-OH	CO-NH
	55.17	31.41	5.42	8
N(%)	N-H/C-N	Amide-N	HN-C(O)O/N-C=C	
	4.28	66.68	29.04	
O(%)	C-O-C/C-OH	O-C=O/O=C-N		
	53.08	46.92		
P(%)	P-C/P-O	C-P-O	$(PO_4)^{3-}$	
	67.41	28.43	4.16	
S(%)	sulfide	oxgsulfide	Sulphate or sulfone	sulfate
	32.32	6.96	55.45	5.27

Table S2. The content of secondary structure.

Untreatedball milling (%)	30°C	50°C	70°C	90°C	110°C	130°C	150°C
β-sheet	33.15	32.64	32.29	32.00	31.64	31.46	31.35
Random coil	11.29	11.20	11.10	10.98	10.90	10.88	10.83
α-helices	14.18	14.33	14.34	14.34	14.35	14.45	14.50
β-turn	41.37	41.83	42.27	42.68	43.11	43.22	43.32
Treated ball milling (%)	30°C	50°C	70°C	90°C	110°C	130°C	150°C
β-sheet	29.27	29.47	29.31	29.43	29.98	30.62	29.06
Random coil	11.51	11.43	11.47	11.48	11.52	11.60	11.36
α-helices	13.23	13.15	13.23	13.07	13.08	13.09	12.97
β-turn	46.00	45.95	45.99	46.02	45.42	44.69	46.62

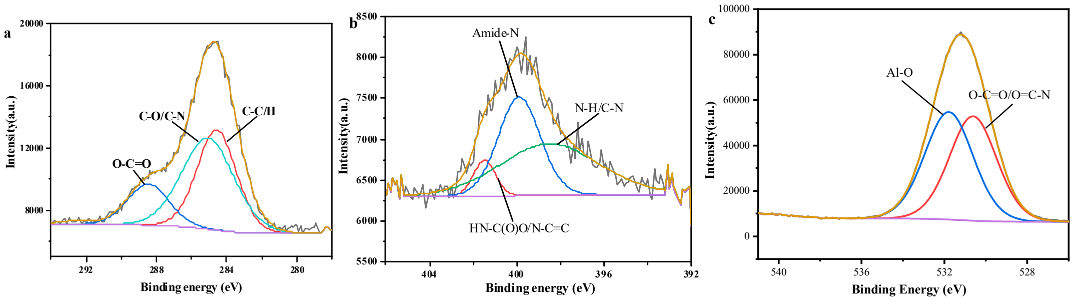

Figure S1. The results of XPS Al@PBSP.

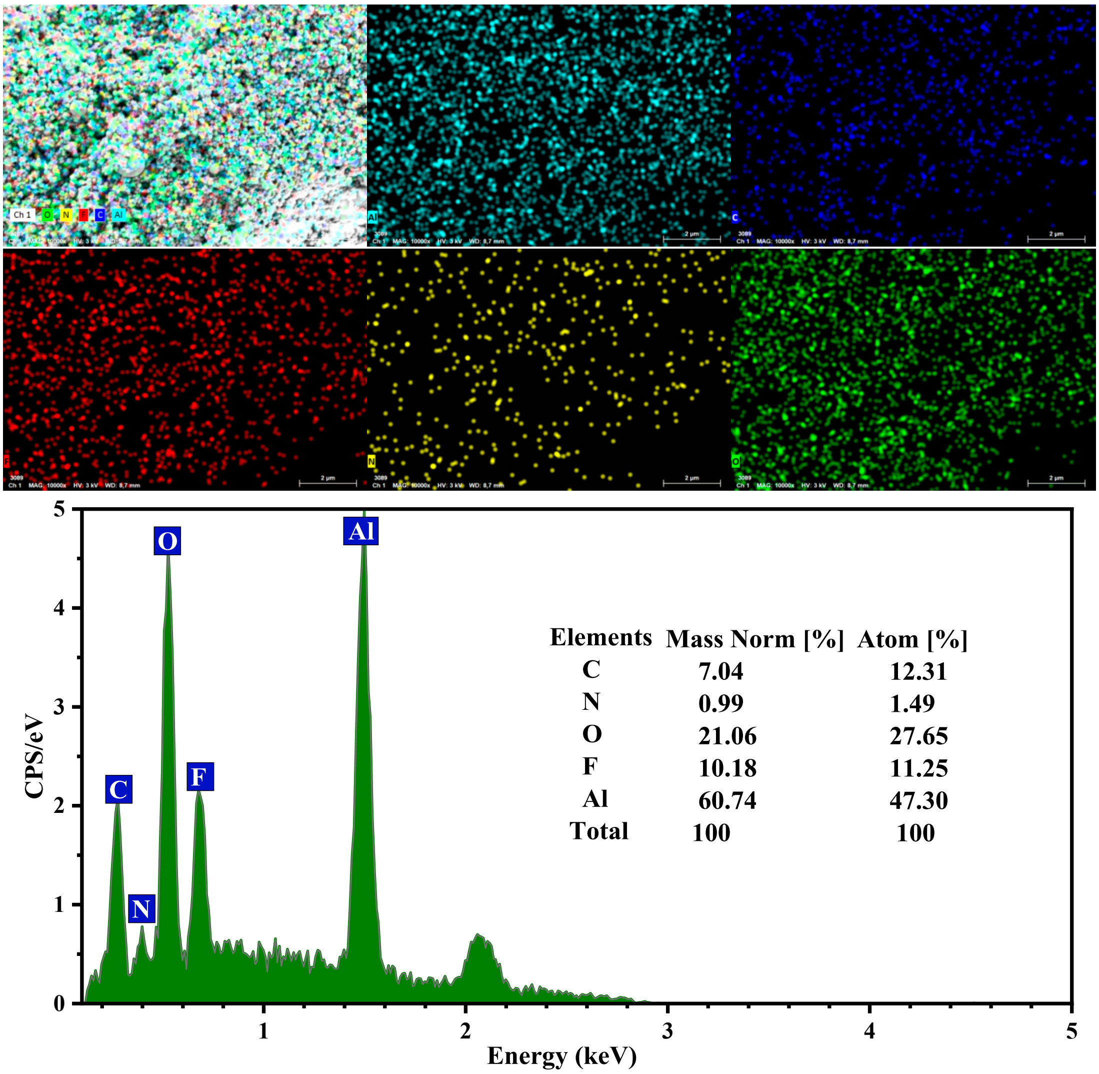

Figure S2. n-Al@ABSE@PVOF SEM-EDS

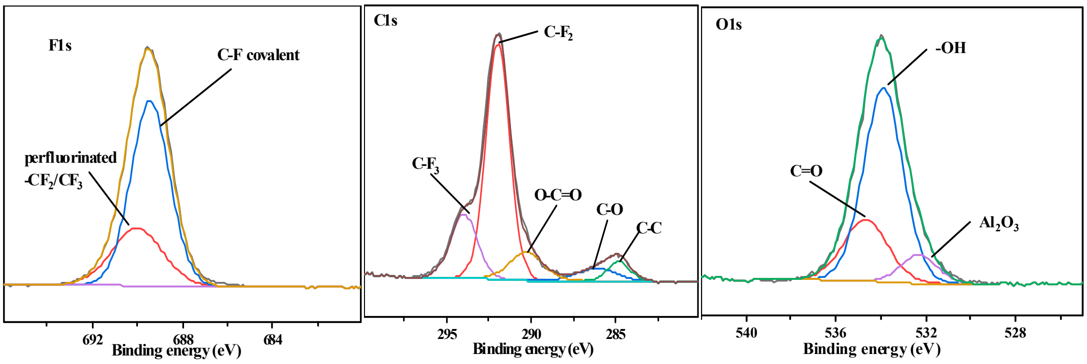

Figure S3. The results of XPS Al@PBSP/PFOA.
